# The Role of Angiotensin II in Poisoning-Induced Shock—a Review

**DOI:** 10.1007/s13181-022-00885-4

**Published:** 2022-03-08

**Authors:** Andrew Chen, Anselm Wong

**Affiliations:** 1 Melbourne Medical School, University of Melbourne, Melbourne, VIC Australia; 2 Department of Medicine, School of Clinical Sciences at Monash Health, Monash University, Melbourne, VIC Australia; 3 Department of Critical Care, University of Melbourne, Melbourne, VIC Australia

**Keywords:** Angiotensin II, Shock, Drug poisoning, Overdose

## Abstract

**Background:**

Shock in drug poisoning is a life-threatening condition and current management involves fluid resuscitation and vasopressor therapy. Management is limited by the toxicity of high-dose vasopressors such as catecholamines. Clinical trials have shown the efficacy of angiotensin II as an adjunct vasopressor in septic shock. The aim of this review is to assess the use of angiotensin II in patients with shock secondary to drug overdose.

**Methods:**

Medline (from 1946), Embase (from 1947) and PubMed (from 1946) databases were searched until July 2021 via OVID. Included studies were those with shock due to drug poisoning and received angiotensin II as part of their treatment regimen. Of the 481 articles identified, 13 studies (case reports and scientific abstracts) were included in the final analysis with a total of 14 patients. Extracted data included demographics, overdose drug and dosage, angiotensin II dosage, time of angiotensin II administration, haemodynamic changes, length of hospital stay, mortality, complications, cardiac function and other treatment agents used.

**Results:**

Thirteen studies were included consisting of 6 case reports, 6 scientific abstracts and 1 case series. Overdose drugs included antihypertensives (*n* = 8), psychotropics (*n* = 4), isopropanol (*n* = 1) and tamsulosin (*n* = 1). Out of a total of 14 patients, 3 patients died. Ten patients had their haemodynamic changes reported. In terms of MAP or SBP changes, three patients (30%) had an immediate response to angiotensin II, four patients (40%) had responses within 30 min, one patient (10%) within two hours and two patients (20%) did not have their time reported. Two patients were shown to have direct chronotropic effects within 30 min of angiotensin II administration. The median hospital stay for patients was 5 days (IQR = 4). The time from overdose until angiotensin II administration ranged from 5 to 56 h. Other vasopressors used included phenylephrine, noradrenaline, adrenaline, vasopressin, dobutamine, dopamine, methylene blue and ephedrine. A median of 3 vasopressors were used before initiation of angiotensin II. Twelve patients received angiotensin II as their final treatment.

**Conclusions:**

Angiotensin II may be useful as an adjunct vasopressor in treating shock secondary to drug poisoning. However, the current literature consisted of only very low-quality studies. To truly assess the utility of angiotensin II use in drug-induced poisoned patients, further well-designed prospective studies are required.

**Supplementary Information:**

The online version contains supplementary material available at 10.1007/s13181-022-00885-4.

## Introduction

Shock is a life-threatening condition where low blood pressure leads to impaired organ perfusion [1]. Maintaining an adequate mean arterial pressure (MAP) is crucial for organ protection as the duration of hypotension is associated with increased organ injury [2]. If left untreated, it can lead to death from multiple organ failure and impaired oxygen utilisation [3]. Current management of shock involves aggressive fluid resuscitation and vasopressor therapy [4]. Vasopressors such as catecholamines and vasopressin are available for clinicians to use as part of conventional management of peripherally shocked patients [5]. Currently, there is no evidence that one vasopressor is superior over another in terms of mortality, with the exception of dopamine, which has a higher risk of arrhythmia and 28-day mortality when compared to noradrenaline [1].

Angiotensin II is an endogenous octapeptide that is a major component of the renin–angiotensin–aldosterone system (RAAS) [6]. It regulates blood pressure through a variety of mechanisms including smooth muscle vasoconstriction of peripheral vessels, reabsorption of water via anti-diuretic hormone (ADH) and potentiating the release of aldosterone [7]. Angiotensin-converting enzyme type-1 (ACE-1) is present in the lungs and aids the cleavage of angiotensin I into angiotensin II [8]. To exert its haemodynamic effect, angiotensin II stimulates AT-1 receptors in the peripheral vasculature [9]. Intravenous angiotensin II has a half-life of less than one minute in circulation and 15 to 30 min in tissue [10]. Clearance is independent of hepatic or renal function but the official prescribing information notes that formal studies have not examined its metabolism [11].

In certain circumstances, management of shock in poisoned patients may be refractory to standard treatment. For example, combined overdoses of dihydropyridine calcium channel blockers with angiotensin receptor blockers (ARBs) or angiotensin-converting enzyme inhibitors (ACEIs) can lead to a refractory shock [12]. Given the wide range of indications for antihypertensives such as beta blockers, as well as their relatively narrow therapeutic index, the potential for death following overdose is significant [13]. Therefore, there may be a role for angiotensin II in cases of drug-induced shock.

Two recent randomised controlled trials have shown the effectiveness of angiotensin II in patients with distributive and vasodilatory shock that did not respond to conventional management [14, 15]. The largest randomised controlled trial (ATHOS-3) to date found that a significantly greater number of patients who received angiotensin II reached primary endpoints with respect to MAP at the 3-h mark when compared to placebo [15]. This led to its approval by the US Food and Drug Administration (FDA) in 2017 and European Medicines Agency (EMA) in 2019 for the treatment of hypotension in patients with septic or other distributive shock [16, 17].

Previous reviews have focused on angiotensin II and its use in the context of various forms of shock including cardiogenic, septic, haemorrhagic and neurogenic forms [18–20]. However, there has been little focus on shocked patients secondary to drug poisoning. This review aims to explore the use of angiotensin II as an adjunct vasopressor in the context of circulatory shock secondary to drug-induced toxicity.

## Methodology

A systematic review was performed across several databases including Medline (from 1946), Embase (from 1947) and PubMed (from 1946) up until 28th July 2021. The search strategy was developed based on the search strategies of similar systematic reviews investigating shock and drug poisoning [13, 18]. A detailed search strategy is outlined in Tables 2 and 3 in the Appendix.

### Search Terms

Medical Subject Headings (MeSH) terms were used in OVID to search Medline and Embase databases. Search terms included ‘exp Renin-angiotensin system’ OR ‘exp angiotensin II’ AND ‘exp shock’ OR ‘exp hypotension’ OR ‘exp vasodilation’ AND ‘exp drug overdose’ OR ‘exp poisoning’. MeSH terms were appropriately translated from MEDLINE to Embase. An additional search of PubMed using the terms angiotensin II and overdose was performed.

### Inclusion and Exclusion Criteria

For inclusion in the analysis, study subjects must have received angiotensin II as an adjunct or secondary treatment for shock. All studies including controlled trials, observational studies, case reports and abstracts from scientific meetings without date restrictions were included. References of included studies were also explored for additional articles. Studies were excluded if they did not include patients who had drug-induced toxicity from overdose. Two investigators agreed on the final inclusion of papers from the literature search.

### Outcome Measures

The primary outcome was mortality. Secondary outcomes included the change in haemodynamic parameters such as MAP or systolic blood pressure (SBP) in association with angiotensin II, length of stay in hospital and reduction of overall catecholamine requirements.

### Quality Assessment

The GRADE (Grading of Recommendations, Assessment, Development and Evaluations) method was used to assess the quality of the studies to assist any future guidelines on poisoning management [21]. GRADE provides a reproducible and transparent framework for grading evidence with specific domains, which increase or decrease the level of confidence in the evidence [22]. Domains which decrease quality of evidence include risk of bias, imprecision, inconsistency, indirectness and publication bias [23]. There are domains which increase the quality of evidence, but these situations are rare [24].

## Results

**Fig. 1:**
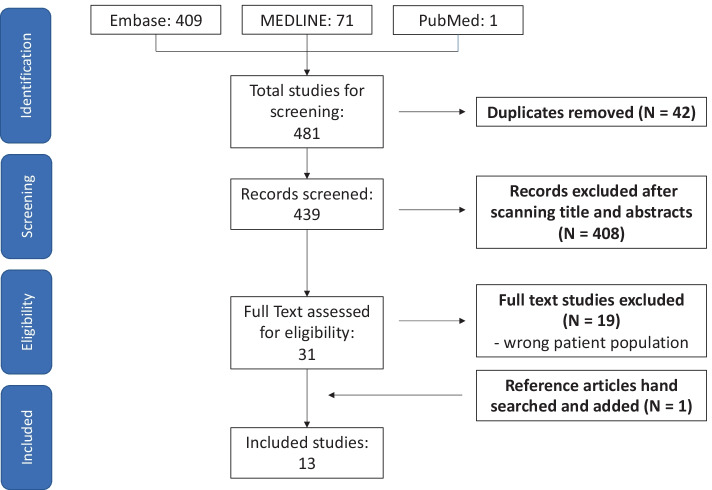
PRISMA diagram representing the search and screen process

The initial search was performed without language restrictions or limitation to research design, which yielded 481 articles, of which 42 (9%) were duplicates. The remaining 439 title and abstracts were screened. This resulted in 31 (7%) full-text relevant articles being identified. Out of these studies, 19 studies were excluded as they did not include the correct patient population (shock induced by overdose). Twelve articles remained after review of full texts. An additional study was found via a secondary search through the references of included studies [25, 26]. Of the remaining 13 articles, six were case reports, six were scientific abstracts and one was a case series (defined as two or more cases of drug overdose reported together). The article selection process is detailed in Fig. 1.

### Angiotensin II in Overdose Patients

From the literature search, six case reports, six scientific abstracts and one case series revealed 14 patients with refractory shock due to drug overdose who were administered angiotensin II as an adjunct part of their treatment. There were no randomised controlled trials or observational studies describing angiotensin II as a treatment in poisoned patients. Patients were hypotensive due to antihypertensives (*n* = 8), psychotropics (*n* = 4), isopropanol (*n* = 1) and tamsulosin (*n* = 1). The antihypertensives included amlodipine, carvedilol, lisinopril, spironolactone, hydralazine, furosemide, hydrochlorothiazide, diltiazem, benazepril, enalapril and verapamil. Psychotropics included doxepin, clozapine, diazepam, risperidone, valproic acid, bupropion, venlafaxine and temazepam. Patients were aged from 18 to 65, comprising six females (43%) and eight males (57%). The main outcome measures that were reported included mortality and changes in MAP/SBP. Out of the 14 patients, three patients (21%) died despite receiving angiotensin II. Ten patients had positive haemodynamic responses reported in terms of MAP or SBP. Eight patients had their response time recorded, all of which occurred within two hours. Two patients were reported to have positive chronotropic effects following angiotensin II administration. Twelve patients received angiotensin II as their final treatment. Nine patients had their initiation of angiotensin II post overdose reported. One patient had the time of initiation reported after receiving first-line vasopressors. The most common disposition was being discharged to psychiatric team. A detailed summary is listed in Table 1.

**Table 1:** Summary of studies

Study	Age (years)	Sex	Echocardiography results	SVR reported	Drug	Poisoning dose	Ang II dose	Time post overdose Ang II administered	Change following Ang II	Hospital stay	Mortality	Complications post Ang II administration	Pre-angiotensin II treatment
1. Ferdowsali et al. (2020) [27]	42	F	NR	595 dyn/s per cm^−5^ before Ang II	TCA doxepin	21 tablets of unknown strength	10 ng/kg/min for ~ 2 days	NR	Within 5 min, MAP ↑ to 67 mmHg Phenylephrine, noradrenaline and adrenaline were weaned off over the next 3 h	9 days	Died	Encephalopathy Acute respiratory distress syndrome secondary to aspiration pneumonitis	Phenylephrine, noradrenaline and adrenaline (noradrenaline-equivalent) dose: 0.55 μg/kg/min
2. Carpenter et al. (2019) [28]	24	F	EF of 40% (from 25%) 15 min after Ang II	NR	Amlodipine, carvedilol, lisinopril, spironolactone, hydralazine, isosorbide mononitrate, furosemide and aspirin	NR	10 ng/kg/min for ~ 24 h	9 h	Within 15 min, MAP ↑ by 12 mmHg and HR ↑ by 7 beats/min Within 1 h, BP ↑ to 108/50 mmHg Over the next 24 h, adrenaline, Ang II, vasopressin and noradrenaline were weaned off in that order	5 days	Alive	Acute kidney injury on hospital day 2, returning to normal on day 5	Activated charcoal: 50 g Noradrenaline: 30 μg/min Adrenaline: 10 μg/min Vasopressin: 0.04 U/min Glucagon IV: 5 mg Hydrocortisone sodium succinate: 100 mg/8 h
	65	M	EF of 45–50% with grade 1 diastolic dysfunction and abnormal septal motion 30 min after Ang II	NR	Carvedilol Amlodipine Lisinopril/hydrochlorothiazide	750 mg (12.5 mg × 60 tablets) 300 mg (10 mg × 30 tablets) 20/25 mg (unknown amount)	10 ng/kg/min for ~ 3 days	5 h	Within 30 min, MAP ↑ to 66 mmHg (from 59) and HR ↑ to 61 beats/min (from 47) After 3 days, all treatment was weaned off	3 days	Alive	No complications	Glucagon IV: 2 mg Noradrenaline: 40 μg/min Adrenaline: 11 μg/min Hyperinsulinemia-euglycemia: IV bolus of 1 U/kg followed by infusion at 1 U/kg/h
3. Guo et al. (2019) [29]	25	M	NR	NR	Metformin, valproic acid, risperidone and trazodone	NR	80 ng/kg/min for ~ 17 h	NR	Vasopressin was ceased upon starting Ang II Within 10 h, phenylephrine infusion was weaned off Within 17 h, all treatment was stopped	5 days	Alive	NR	High doses of noradrenaline, adrenaline, vasopressin and phenylephrine L-carnitine Continuous renal replacement therapy
4. Trilli et al. (1994) [30]	45	M	NR	339 dyn/s per cm^−5^ before Ang II	Lisinopril	25 mg	8.5–9 μg/min for ~ 120 h	56 h	Immediately, SBP ↑ to 70 mmHg (from 46) After 48 h, dopamine was down titrated	10 days	Died	No complications from hypotensive crisis Day 10 developed catheter-related sepsis	Dobutamine: 20 μg/kg/min Dopamine: 20 μg//kg/min Noradrenaline: 14.5 μg/min
5. Quinn et al. (2021) [31]	50	M	NR	Pulmonary artery catheterisation revealed low cardiac indices (< 2.3), low central venous oxygen saturations (< 70%), high filling pressures and low systemic vascular resistance before Ang II	Diltiazem, bupropion and venlafaxine	NR	35 ng/kg/min for 18 h	NR	Immediately MAP sustained above 65 (from 40 s-50 s) VA-ECMO was deemed unnecessary After 72 h, all treatment stopped	3 days	Alive	NR	Noradrenaline: 300 μg/min Vasopressin 0.04 U/min Adrenaline: 20 μg/min Dopamine: 2 μg/min One-to-one diastolic augmentation via intra-aortic balloon pump
6. Gutierrez et al. (2019) [32]	57	M	NR	NR	Amlodipine and benazepril	NR	20–80 ng/kg/min for 4 days	NR	Within 2 h, adrenaline was weaned off and noradrenaline ↓ by 50% 4 days later, all vasopressors were weaned off	8 + days	Alive	No persistent end-organ failure	Calcium gluconate: 4 g Glucagon: 10 mg High-dose euglycemic therapy: IV insulin 3.5 units/kg/h 20% lipid emulsion: 1.5 mL/kg bolus × 2 Noradrenaline, phenylephrine and vasopressin
7. Jackson et al. (1993) [26]	44	M	NR	NR	Enalapril Verapamil Temazepam	600 mg (20 mg × 30 tablets) 7200 mg (240 mg × 30 tablets) 100 mg (10 mg × 10 tablets)	3–18 μg/kg/min for 3 h 5 μg/kg/min for final 2 h	8 h after other vasopressors were given	SBP sustained to above 100 mmHg within 2 h Urine output ↑ > 100 mL/hour (from 20 mL/hour) Conversion from junctional to sinus rhythm	NR	Alive	NR	Calcium gluconate, ephedrine and atropine Dopamine: 3 μg/kg/min Adrenaline: 0.02 μg/kg/min
8. Eisenstat et al. (2020) [33]	65	M	NR	NR	Tamsulosin Genvoya (elvitegravir, cobicistat, emtricitabine, tenofovir) Darunavir	12 mg (0.4 mg × 30 tablets) 150–150-200–10 mg (30 tablets) 24,000 mg (800 mg × 30 tablets)	NR	~ 10 h	NR	NR	Died	Pulseless electrical activity arrest	Noradrenaline, phenylephrine, vasopressin
9. Newby et al. (1995) [25]	46	F	Good left ventricular function without pericardial effusion before Ang II	1724 dyn/s per cm^−5^ before Ang II	Enalapril Strong lager	140–200 mg (10 mg × 14–20 tablets) 5 pints	22 ng/kg/min for 30 h	36–48 h	BP ↑ to 110/70 mmHg (from 80/50) Urine output ↑ to 1500 mL in 4 h (from anuria)	NR	Alive	No complications	Dopamine: 2.5 μg/kg/min Noradrenaline: bolus 1 mg over 2 min
10. Ulici et al. (2021) [34]	18	F	EF of 65–70% with HR 130 (unknown whether before/after Ang II)	NR	Amlodipine Carvedilol, furosemide and hydrochlorothiazide	300 mg 30 tabs of unknown strength (25 mg hydrochlorothiazide)	40 ng/kg/min	20 h	Noradrenaline, high-dose insulin, Ang II, vasopressin, phenylephrine required to maintain MAP above 50 mmHg Vasopressors weaned off after 5 days	9 + days	Alive	Good neurological outcome	Calcium gluconate: 2 mg Insulin: 9 units/kg/hour Noradrenaline: 0.45 μg/kg/min Vasopressin: 2.4 units/min Phenylephrine Methylene blue: bolus 1 mg/kg and infusion 2 mg/kg/hour VA-ECMO
11. Wieruszewski et al. (2020) [35]	39	M	Hyperdynamic ventricles with grossly preserved EF and no pericardial effusion before Ang II	NR	Clozapine	11 g	20 ng/kg/min for 8 h 30 ng/kg/min for final 8 h	~ 8 h	Within minutes, MAP ↑ 66 mmHg (from 50–55) No change in urine output (consistent oliguria) after 6 h After 8 h, noradrenaline and adrenaline down titrated to 0.8 and 0.55 μg/kg/min respectively After 16 h, noradrenaline and vasopressin down titrated to 0.1 μg/kg/min and 0.04 U/min respectively All vasopressors stopped after 18 h	5 days	Alive	Persistent oliguria and acidosis—leading to renal replacement therapy Recovered renal function at discharge	Noradrenaline: 1 μg/kg/min Adrenaline: 1 μg/kg/min Vasopressin: 0.08 U/min Stress-dose corticosteroids
12. Tovar et al. (1997) [36]	34	F	NR	NR	Enalapril Ramipril Amlodipine Nitrendipine	200 mg 110 mg 110 mg 600 mg	5–15 μg/min for 24 h	7.5 h	Almost immediately, SBP rose from 50 to 100 mmHg Noradrenaline reduced to 10 μg/min Immediate improvement in diuresis output and disappearance of cardiac failure signs After 72 h, all vasopressors treatment stopped	4 + days	Alive	Patient totally recovered	IV calcium gluconate: 1000 mg Dopamine: 4.9 μg/kg/min Noradrenaline: 60 μg/min
13. Chieng et al. (2021) [37]	44	F	Hyperdynamic left ventricle before Ang II	NR	Isopropanol Diazepam	Up to 500 mL of isopropanol 60% and chlorhexidine 0.5% 5 mg	20–70 ng/kg/min for ~ 4 h	12.5 h	After 3.5 h, methylene blue and vasopressin weaned off	5 + days	Alive	Remained well	Noradrenaline: 90 μg/min Vasopressin: 0.04 units/min Methylene blue: 1 mg/kg/hour CVVHDF

*CO*, cardiac output; *CVVHDF*, continuous venovenous haemodiafiltration; *echo*, echocardiogram; *EF*, ejection fraction; *F*, female; *HR*, heart rate; *IV*, intravenous; *M*, male; *MAP*, mean arterial pressure; *NR*, not reported; *N/A*, not applicable; *SBP*, systolic blood pressure; *SVR*, systemic vascular resistance; *TCA*, tricyclic antidepressant; *VA-ECMO*, venoarterial extracorporeal membrane oxygenation

### Risk of Bias

As expected, all studies were classified as ‘very low’ quality using the GRADE criteria due to the nature of the study design (case reports and abstracts). Risk of bias was high due to the considerably high risk of publication, confounding, confirmation and observer bias. A detailed table is listed in Table 4 in the Appendix.

## Discussion

The quality of evidence examining angiotensin II in drug-induced poisoning was very low as per the GRADE methodology. There were often confounding factors due to the institution of multiple treatments involving multiple medications. No studies examined differences in length of hospital stay or compared treatments.

### Mortality

Out of the 14 patients across the 13 studies, three patients (21%) died despite restoration of blood pressure after angiotensin II infusion. These included poisoning from lisinopril, tamsulosin and doxepin. Although initially recovering from their period of hypotension, these deaths were due to complications from catheter-related sepsis (with lisinopril) and a combination of encephalopathy and ARDS (with doxepin). Details of the third fatality were lacking.

Historically, septic shock that was refractory to conventional vasopressors had mortality rates as high as 90% [38]. Refractory shock is common and despite advances in therapy, the mortality of patients with shock remains as high as 30–50% [39]. The ATHOS-3 trial did not show any short or long-term mortality benefit in the angiotensin II treatment group when compared to the placebo group [15]. However, it did demonstrate that the angiotensin I/angiotensin II ratio was predictive of both blood pressure and mortality [15]. Eighty percent of patients in this trial had shock due to sepsis, with a median age of 64. This is in contrast to poisoned patients who are usually younger and were less likely to have premorbid comorbidities.

### Mean Arterial Pressure, Systolic Blood Pressure, Heart Rate and Treatment Times

Maintaining adequate perfusion is crucial in drug-induced hypotension to allow for the clearance of the toxic metabolites [29, 31]. Other agents that were used included phenylephrine, noradrenaline, adrenaline, vasopressin, dobutamine, dopamine, methylene blue and ephedrine. Ten patients had their haemodynamic changes reported, with eight patients having their response time in relation to MAP or SBP improvement described. In terms of MAP or SBP changes, three patients (30%) had an immediate response to angiotensin II, four patients (40%) had responses within 30 min, one patient (10%) within two hours and two patients (20%) did not have their time reported. Six patients (60%) had their MAP reported and five patients achieved a MAP of > 65 mmHg after angiotensin II treatment, with one patient reaching a MAP of > 50. SBP changes were reported in the remaining four patients (40%), with three patients achieving a SBP of > 100 and one patient achieving a SBP of > 70. Across all 14 patients, 12 patients (86%) had their treatment times reported, which ranged from 4 to 120 h.

Angiotensin II does not typically exhibit chronotropic or inotropic effects [4]. One case series reported direct chronotropic changes, where two patients had an increase in heart rate within 30 min of angiotensin II administration [28]. Busse et al.’s recent literature review demonstrated the successful use of angiotensin II in patients with shock from various aetiologies including distributive, cardiogenic and haemorrhagic [18]. However, the evidence consisted of mostly low-quality studies such as case reports and was subject to publication bias [18]. Angiotensin II has been approved for use only in cases of vasodilatory shock refractory to standard vasopressor therapy [11]. In our review, angiotensin II was used in a heterogeneous group of drug-induced shock which may have included cardiogenic or vasoplegic shock. Only a few studies mentioned cardiac function investigations.

### Other Cardiovascular Support Treatments

Nine patients (64%) had their weaning period of first-line vasopressors reported, which ranged from 2 to 48 h. This finding is consistent with ATHOS and ATHOS-3 trials where all patients receiving angiotensin II had a reduction in catecholamine requirements [14, 15]. Additionally, the vasopressor dose required to maintain MAP is highly predictive of short-term mortality in critically ill patients [40–43]. However, vasopressor reductions that occur across days may be due to a combination of both the elimination of toxic metabolites and the effect of angiotonin II itself. The elimination of drugs is dependent on the half-life of the drug and the amount ingested.

There have been numerous case reports describing the use of venoarterial and venovenous ECMO for the treatment of drug-induced shock from antihypertensives and anti-depressants [44–46]. ECMO in the clinical setting should be considered in patients with cardiorespiratory failure who deteriorates rapidly despite maximal conventional treatment [47]. During ECMO therapy, angiotensin II levels are reduced as blood bypasses the lung where angiotensin I is converted into angiotensin II [48]. In this review, one patient exhibited successful treatment with ECMO after receiving angiotensin II [34]. Another patient did not require ECMO after angiotensin II was administered [31].

### Limitations

This systematic literature review has various limitations. Primarily, all literatures were of very low evidence as only case reports, case series and scientific abstracts were found. The assessment of mortality was limited due to the small nature of the same size. As with all case reports, a comparator population was not available, which makes it difficult to establish whether patients would have improved with standard treatment. Finally, this review is limited by reporting bias as reporting was not standardised and improvement in relation to timing was not always stated. Further randomised controlled trials with larger sample sizes are needed to address these limitations.

## Conclusion

Angiotensin II may be useful as an adjunct vasopressor in treating shock secondary to drug poisoning. However, the current literature consisted of only very low-quality studies. To truly assess the utility of angiotensin II use in drug-induced poisoned patients, further well-designed prospective studies are required.

## Electronic supplementary material

Below is the link to the electronic supplementary material.

Supplementary file1 (PNG 684 KB)

## Appendix

**Table 2:** Search Strategy: Ovid MEDLINE(R) and Epub Ahead of Print, In-Process, In-Data-Review & Other Non-Indexed Citations and Daily < 1946 to July 28, 2021 >

1	exp Renin-Angiotensin System/ or exp Angiotensin II/	52,515
2	(hypertensin or angiotonin or ‘angiotensin II’ or ‘angiotensin 2’).tw	51,646
3	1 or 2	74,225
4	exp Shock, Cardiogenic/ or exp Shock/ or exp Shock, Hemorrhagic/ or exp Shock, Septic/	79,131
5	exp Hypotension/ or exp Vasodilation/	60,994
6	vasodilatory shock.mp	295
7	4 or 5 or 6	137,854
8	exp Poisoning/	161,837
9	exp Opiate Overdose/ or exp Drug Overdose/	12,500
10	exp Chemically-Induced Disorders/	515,125
11	(overdose* or poisoning or toxicity or intoxication or ingestion* or adverse effect* or medication error*).tw	732,215
12	8 or 9 or 10 or 11	1,161,793
13	3 and 7 and 12	71

**Table 3:** Search Strategy: Embase Classic + Embase < 1947 to 2021 Week 29 >

1	exp renin angiotensin aldosterone system/ or exp angiotensin II/	71,589
2	(hypertensin or angiotonin or ‘angiotensin II’ or ‘angiotensin 2’).tw	67,857
3	1 or 2,108,545	108,545
4	exp shock/ or exp cardiogenic shock/ or exp hemorrhagic shock/ or exp septic shock/	157,924
5	exp hypotension/ or exp vasodilatation/	250,500
6	vasodilatory shock.mp	476
7	4 or 5 or 6	391,279
8	exp intoxication/	439,832
9	exp drug overdose/	31,108
10	exp chemically induced disorder/	121,393
11	(overdose* or poisoning or toxicity or intoxication or ingestion* or adverse effect* or medication error*).tw	1,031,784
12	8 or 9 or 10 or 11	1,415,370
12	3 and 7 and 12	409

**Table 4:** Quality assessment

Authors	GRADE factors							
	Study design	Risk of bias	Imprecision	Inconsistency	Indirectness	Publication bias	Other considerations	Overall quality
Ferdowsali et al., 2020 [27]	Case report	Serious	-	-	Not serious	Serious	None	Very low
Carpenter et al., 2019 [28]	Case report	Serious	-	-	Not serious	Serious	None	Very low
Guo et al., 2019 [29]	Scientific abstract	Serious	-	-	Not serious	Serious	None	Very low
Trilli et al., 1994 [30]	Case report	Serious	-	-	Not serious	Serious	None	Very low
Quinn et al., 2021 [31]	Scientific abstract	Serious	-	-	Not serious	Serious	None	Very low
Gutierrez et al., 2019 [32]	Scientific abstract	Serious	-	-	Not serious	Serious	None	Very low
Eisenstat et al., 2020 [33]	Scientific abstract	Serious	-	-	Serious	Serious	None	Very low
Jackson et al., 1993 [26]	Case report	Serious	-	-	Not serious	Serious	None	Very low
Newby et al., 1995 [25]	Case report	Serious	-	-	Not serious	Serious	None	Very low
Ulici et al., 2021 [34]	Scientific abstract	Serious	-	-	Not serious	Serious	None	Very low
Wieruszewski et al., 2020 [35]	Case report	Serious	-	-	Not serious	Serious	None	Very low
Tovar et al., 1997 [36]	Case report	Serious	-	-	Not serious	Serious	None	Very low
Chieng et al., 2021 [37]	Case report	Serious	-	-	Not serious	Serious	None	Very low
